# Evaluation of the predictors of successful sperm retrieval of micro-TESE in cases with mosaic Klinefelter versus cases with non-mosaic Klinefelter: a prospective case series study

**DOI:** 10.1186/s12610-025-00265-2

**Published:** 2025-05-19

**Authors:** Amr Elahwany, Fatma A. Elrefaey, Hisham Alahwany, Hesham Torad, Sameh Fayek GamalEl Din, Rashad Mohammed Saeed Dawood, Mohamed Wael Ragab, Ahmed Fawzy Megawer

**Affiliations:** 1 Department of Andrology and STDs Kasr Al-Ainy, Faculty of Medicine Cairo University, Al-Saray Street, El Manial, Cairo, 11956 Egypt; 2 Nile IVF Center, Cairo, Egypt; 3 Department of Clinical Pathology, National Cancer Institute, Cairo, Egypt; 4 Department of Urology, Kasr Alainy Faculty of Medicine, Cairo University, Cairo, Egypt; 5 Ministry of Health, Sanaae, Republic of Yemen; 6 Dar Alnokhba fertility center, Cairo, Egypt, Cairo, Egypt

**Keywords:** Mosaic Kleinfelter syndrome, Azoospermia, Micro-TESE

## Abstract

**Background:**

We evaluated the predictors of eventful microsurgical testicular sperm extraction (micro-TESE) from infertile men with Klinefelter syndrome (KS).

**Results:**

The mean age of the patients was 32.4 ± 6.3 years. The mean serum levels of follicle stimulating hormone (FSH), luteinizing hormone (LH), total testosterone (TT), estradiol (E2) and prolactin were 34.38 ± 14.66, 18.92 ± 6.54, 3.18 ± 2.08, 28.2 ± 10, 11.56 ± 5.09, respectively. The mean right (Rt) testicular and left (Lt) testicular volumes were 2.17 ± 0.83 ml, 2.2 ± 0.89 ml, respectively. Mosaic KS patients showed highly significant TT compared to non-mosaic KS patients. Twenty-six patients out of 50 patients (52%) showed mature sperm in wet preparation, whereas the sperm retrieval rate (SRR) of the patients with mosaic and non-mosaic KS was (57.1) % and (32.1) %, respectively. SR was significantly associated with testicular volume > 2 ml, total testicular volume > 5 ml and LH < 21.29 IU/L (p 0.007, 0.005, 0.044, respectively). FISH testing results showed that higher 46xy and lower 47xxy were significantly associated with successful sperm retrieval (p 0.014, 0.015, respectively). Rt and Lt testicular volumes, total testicular volume, LH and FISH could significantly predict successful SR. No statistically significant correlations were found between micro-TESE and age, serum FSH, serum TT, E2, prolactin. Further, receiver operation characteristic (ROC) curve showed Rt and Lt testicular volumes and total testicular volume and LH level and 46xy could significantly predict successful SR with p 0.007, 0.007, 0.005 and 0.044 and 0.015, respectively.

Moreover, the cutoff point and sensitivity and specificity and positive and negative predictive values for Rt and Lt testicular volumes were as follows 2 ml, 73.1%, 61.4%, 52.78, 79.41, 2 ml, 76.9%, 57.8%, 51.28 and 80.65, respectively. While these values for total testicular volume were as follows 5.255 ml, 61.5, 75, 59.26 and 76.74, respectively. Furthermore, these values for LH and 46xy were as follows 17 IU/l, 73.1%, 50%, 46.34, 75.86, 16.35, 84.6, 50, 50 and 84.6, respectively.

**Conclusions:**

Patients with mosaic KS had higher rates of SRR compared to non-mosaic KS.

## Résumé

**Contexte:**

nous avons évalué les facteurs prédictifs d'une extraction microchirurgicale testiculaire de spermatozoïdes (micro-TESE) réussie chez des hommes infertiles atteints du syndrome de Klinefelter (KS).

**Résultats:**

L'âge moyen des patients était de 32,4 ± 6,3 ans. Les taux sériques moyens de l'hormone folliculo-stimulante (FSH), de l'hormone lutéinisante (LH), de la testostérone totale (TT), de l'estradiol (E2) et de la prolactine étaient respectivement de 34,38 ± 14,66, 18,92 ± 6,54, 3,18 ± 2,08, 28,2 ± 10 et 11,56 ± 5,09 UI/ml. Les volumes moyens des testicules droit (Rt) et gauche (Lt) étaient respectivement de 2,17 ± 0,83 ml et 2,2 ± 0,89 ml. Les patients atteints de KS mosaïque présentaient un TT très significatif par rapport aux patients atteints de KS non mosaïque. Vingt-six patients sur 50 (52 %) présentaient des spermatozoïdes matures en préparation humide, tandis que le taux de récupération des spermatozoïdes (SR) des patients atteints de KS mosaïque et non mosaïque était respectivement de 57,1% et 32,1%. La SR était significativement associée à un volume testiculaire > à 2 ml, un volume testiculaire total > à 5 ml et un taux de LH < à 21,29 UI/L (p 0,007, 0,005, 0,044, respectivement). Les résultats du test FISH ont montré que des taux plus élevés de 46XY et plus faibles de 47XXY étaient significativement associés à une récupération réussie de spermatozoïdes (p 0,014, 0,015, respectivement). Les volumes testiculaires Rt et Lt, le volume testiculaire total, la LH et le test FISH pouvaient prédire de manière significative le succès de la SR. Aucune corrélation statistiquement significative n'a été trouvée entre la micro-TESE et l'âge, la FSH sérique, la TT sérique, l'E2 et la prolactine. En outre, la courbe ROC (receiver operation characteristic) a montré que les volumes testiculaires Rt et Lt, le volume testiculaire total, le taux de LH et le 46XY pouvaient prédire de manière significative le succès de la SR avec des valeurs p de 0,007, 0,007, 0,005, 0,044 et 0,015, respectivement.

**Conclusions:**

Les patients atteints de KS mosaïque présentaient des taux de SRR plus élevés que ceux atteints de KS non mosaïque.

**Mots clefs:**

syndrome Klinefelter mosaïque, azoospermie, micro-TESE

## Introduction

Klinefelter syndrome (KS) is the most common sex-chromosome abnormality in males. It affects approximately 1 in 500 newborn boys [1]. A recent cohort study had shown that KS or mosaics were the most common karyotype observed (112 men (12.9%) having a non-mosaic 47,XXY karyotype and 8 (0.07%) having mosaic KS) [2]. Moreover, it is the most frequent genetic cause of human infertility occurring in 3% of infertile men [3]. KS is the phenotypic result of a genetic mishap in which an extra X chromosome is present in all (pure KS, 47XXY) or a portion (mosaic KS, 47XXY/46XY) of the somatic and germ cell compartments [4]. About 80% to 85% of cases are due to the congenital numerical chromosome aberration 47XXY [5, 6]. Approximately 15% to 20% of KS men are mosaics, usually with two cell lines: 47XXY/46XY [7]. Focal spermatogenesis and severe oligozoospermia were reported usually in cases of mosaic karyotype whereas azoospermia is shown in most individuals with a pure 47,XXY karyotype in blood cells [8]. The clinical features of KS include hypergonadotropic hypogonadism, gynecomastia, and azoospermia [9]. Great variability is present in the clinical findings, depending on the onset and degree of androgen deficiency experienced by the individual, but most patients with KS have small sized testes and are infertile. Men who had fathered offspring usually had mosaicism [10]. In contrast, men with non-mosaic KS usually have azoospermia and are considered infertile, where testicular tubules become fibrotic and hyalinized [11]. Thus, the tubular lumen gradually obliterates, and germ cells disappear with time [11]. Nevertheless, recent findings have suggested that 47XXY spermatogonia can undergo complete spermatogenesis [11].

Noteworthy, microsurgical testicular sperm extraction (micro-TESE) had been offered to men with KS on hope of finding spermatozoa and had resulted, in many cases, in successful sperm retrieval (SR) [12]. The aim of this case series study was to evaluate the outcome of SR rate (SRR) using micro-TESE as well as the predictors of successful micro-TESE in mosaic KS cases versus non-mosaic KS cases.

## Materials and methods

### Study population

Seventy azoospermic patients who were diagnosed with KS after cytogenic evaluation, were recruited to the andrology outpatient clinic from December 2021 to November 2022. The institutional ethical committee approved the work that conforms to Helsinki declaration 2013 [13]. All patients signed an informed consent prior to enrollment.

### Inclusion criteria of the patients

Patients complained of infertility and their investigations consistent with KS were recruited during the period where the study was conducted.

### Exclusion criteria of the patients

Azoospermic patients due to causes other than KS were excluded from the study.

All patients were subjected to the following:

The patients underwent history and physical examinations. The testicular volume was measured using ultrasound. Semen analysis was performed twice according to the 5 th edition of the WHO guide lines [14].

All the patients had their serum hormone levels measured using chemiluminescence immunoassay (CLIA) technique, with values in the range: 1.5–14 mIU/ml for follicle stimulating hormone (FSH), 1.5–8 mIU/ml for luteinizing hormone (LH), 2.5–17 ng/ml for prolactin, 2.4–8.3 ng/ml for total testosterone (TT) and 20–47 pg/ml for estradiol (E2) were taken as normal. A fasting morning serum sample for basal hormones determination was obtained prior to the micro‐TESE attempt. All assays were performed using Cobas E411 immunoassay analyzer (Roche Diagnostics GmbH, Mannheim, Germany).

### Cytogenetic analysis

Cytogenetic studies were performed on peripheral blood cells after 72-h culture with phytohemagglutinin stimulation. Chromosome analysis was carried out using standard procedures: the cell cycle was synchronized by incubating it with colcemid solution, cells were then incubated in hypotonic KCl solution and fixed in Carnoy solution. The cell suspension was spread and air-dried on glass slides. GTG or RHG banding were performed. Only 20 cells were analyzed. Mosaicism was detected by the presence of two or more cells populations. A cells population was considered when at least two cells had gained the same chromosome or with identical structural chromosomal abnormalities or at least three cells had lost the same chromosome.

### Barr body analysis

Nuclei from buccal epithelial cells were used for X chromatin Barr body counting. After the patient rinsed his mouth with water, microscope slides were scraped along the buccal mucosa. The cells were spread on the slide and fixed for 10 min in a chloroform carnoy solution.

Then, cells cytoplasm was hydrolyzed in HCl solution at 56 °C. Afterwards, the slides are briefly dipped in blue of Toluidine to color the nuclei. At least 200 epithelial cells at 100 × magnification are microscopically analyzed for the presence of stained Barr bodies. A positive Barr body test in males was defined based on counting > 5% of positive Barr body nuclei.

### Buccal mucosa smears preparation

Oral smears were obtained by scraping the inner cheek epithelium and the buccal mucosa cells were processed according to the method of Garcia-Quevedo et al. [15], with some modifications. The cells were incubated with a 0.035 M KCl hypotonic solution for up to 3 h at 37 °C. After that, the cells were washed three times with the Carnoy’s fixative solution before spreading. Slides were treated with 50% acetic acid solution made in H2O for 1 h at 37 °C to make cells more permeable before FISH procedures. Twenty metaphase cell analysis was performed in conventional karyotype which was increased to 30 cells when mosaicism was suspected. Although some labs might extend to 100 cells in cases of low-level mosaicism, yet the guidelines do not provide clear recommendations on the number of cells needed to rule out low-level mosaicism and our practice agrees with Aiko Otsubo et al. (2023) [16]. Most importantly, in the current study, we confirmed our Karyotyping results with Fish technique which is the most reliable in gender determination.

### FISH procedure

**Fig. 1:**
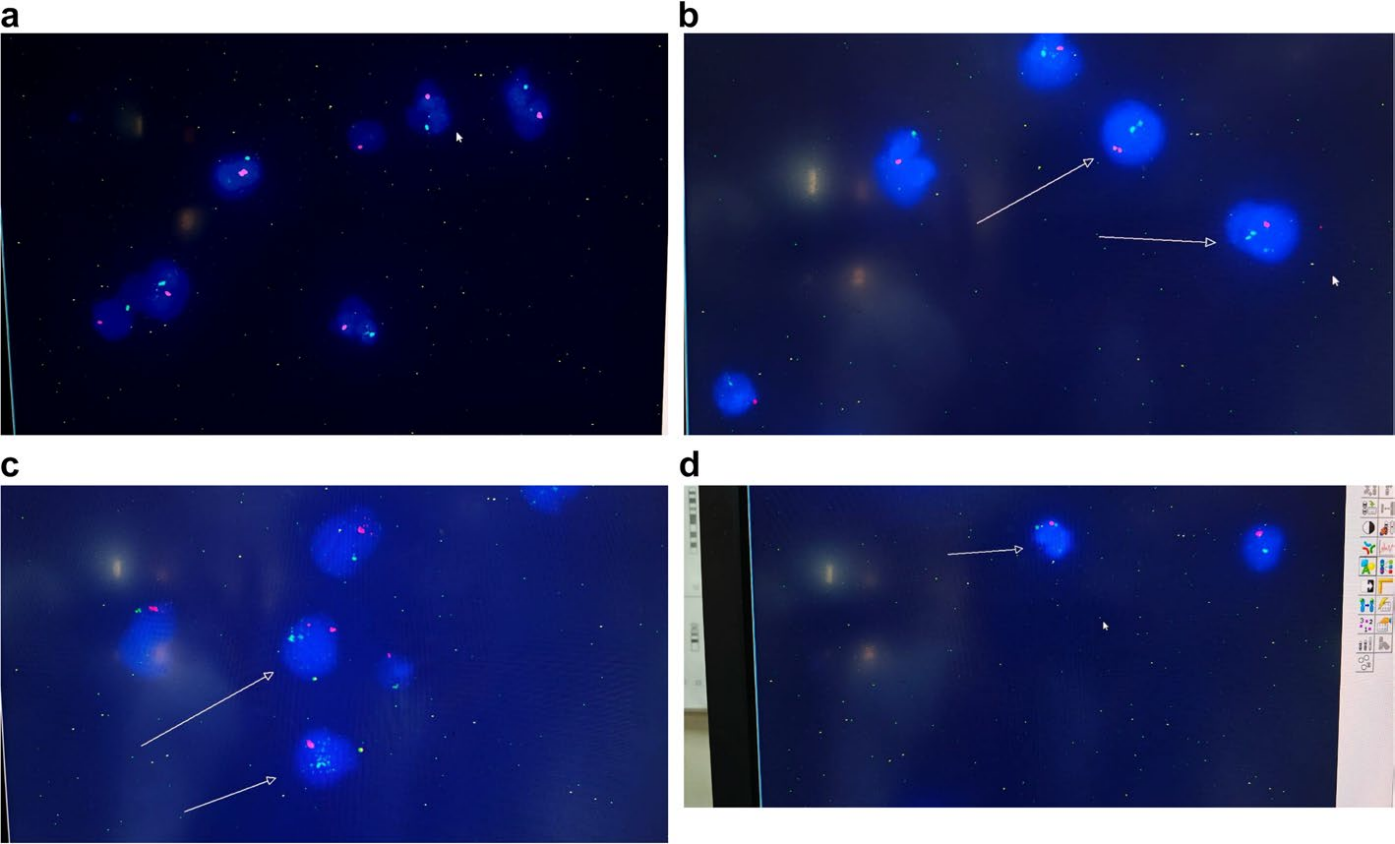
**a**,**b**,**c**,**d** show Bar body analysis

FISH analysis of X and Y chromosomes was performed in both lymphocytes and buccal mucosa cells using centromeric DNA probes for chromosomes X and Y (CEP Y, Spectrum Orange; CEP X, Spectrum Green) according to the manufacturer’s instructions and as described by Lenz et al. [17]. For each participant, at least 100–250 interphase nuclei were examined from both lymphocytes and buccal mucosa cells (Fig. 1 a,b,c,d).

### FISH analysis

For FISH analysis, glass slides were immersed in denaturant solution for 2 min (70% formamid/2 × SSC). After drying the slides, a mixture of fluorescence-labeled probes specific to DXZ1 (CEP X spectrum green, VYSIS) and DYZ3 (CEP Y spectrum orange, VYSIS), were applied. Slides were then hybridized at 37 °C for 15–20 h. After washing, chromosomes were counterstained by DAPI (blue). Slides were examined under a fluorescence microscope equipped with a triple band pass filter. X and Y chromosomes were identified by green and orange fluorescence, respectively. If two signals of the same color, size and intensity were separated by at least one domain, disomy was diagnosed. Mosaicism was assessed in 200 metaphases and nuclei and expressed as the ratio of each karyotype (Fig. 1 a,b,c,d).

### Steps of Micro-TESE

Micro-TESE was performed under spinal or general anesthesia. Under complete aseptic condition, a small skin incision, 1.5–3 cm in the scrotal median raphe. The testicle was delivered, and the operating microscope was brought into the operating field. The tunica albuginea was opened under microscopic magnification.

**Fig. 2:**
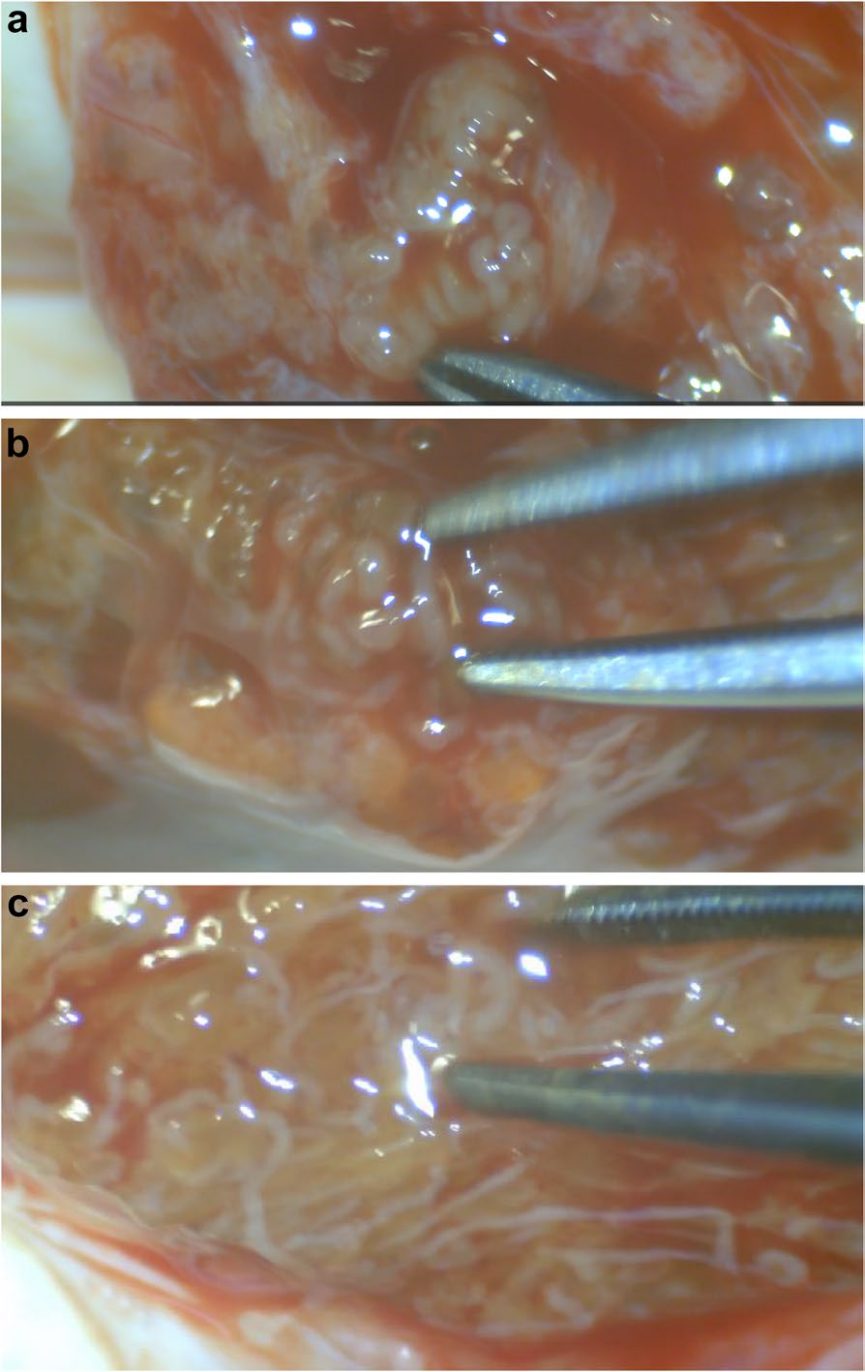
**a**,**b**,**c** show intro-operative photos for the micro-TESE

An equatorial testicular incision was done for all cases. The exposed testicular parenchyma was examined under the operating microscope at 25X to 40X magnification to allow for identification of dilated seminiferous tubules. Jewelers forceps were used for delicate tissue dissection and removal of dilated tubules (Figs. 2 a,b,c). Additionally, dilated tubules were only removed with minimal testicular tissue (Figs. 2 a,b,c). The tubules were minced in petri dish containing 20 micron media droplets (HEPES [4-(2-hydroxyethyl) −1- piperazineethanesulfonic acid]-buffered sperm) using two syringe needles 28G. The micro-biopsies were immediately sent to the IVF laboratory embryologist, to be dissected and examined under high power microscopy to confirm presence of motile or non-motile spermatozoa. The procedure was terminated when several spermatozoa (≥ 5 motile or non-motile spermatozoa) were observed in the micro-biopsies (in the superficial or deeper testis parenchyma) or after complete and thorough examination of the entire testicular parenchyma. The incision was closed in layers, with closure of the tunica albuginea, dartos muscle layer and skin. If fewer than five spermatozoa were identified in the biopsies from the first testicle, the procedure was repeated on the contra-lateral side.

## Results

Mean age of the patients was 32.4 ± 6.3 years (Table 1). Mean serum levels of FSH, LH, TT, E2 and prolactin were 34.38 ± 14.66, 18.92 ± 6.54, 3.18 ± 2.08, 28.2 ± 10, 11.56 ± 5.09, respectively (Table 1). Mean right (Rt) testicular volume was 2.17 ± 0.83 ml, whereas mean left (Lt) testicular volume was 2.2 ± 0.89 ml (Table 1). The clinical characteristics were not statistically significant between the two groups except TT that was significantly higher in mosaic KS (Table 1).

**Table 1:** shows clinical and laboratory characteristics of the participants according to their karyotyping

	Genetic profile				*P* value
	Mosaic		Pure		
	Mean ± SD	Median (range)	Mean ± SD	Median (range)	
Age in years	34.8 ± 7	35.5(20–50)	31.8 ± 6	32(21–48)	0.084
Testis volume right (ml)	2.24 ± 0.75	2.49(0.7–3)	2.15 ± 0.85	2.07(0.7–4.2)	0.697
Testis volume left (ml)	2.17 ± 0.73	2.29(0.92–3.1)	2.21 ± 0.93	2.3(0.66–4.4)	0.826
total testicular volume (ml)	4.42 ± 1.46	4.59(1.62–6.10)	4.36 ± 1.75	4.41(1.41–8.60)	0.994
TT (nmol/l)	4.6 ± 2.45	4.05(2.7–12)	2.83 ± 1.84	2.26(0.14–8.22)	0.001
FSH (IU/l)	32.35 ± 13.84	30.8(11–62.9)	34.88 ± 14.93	34.5(9–76)	0.665
LH (IU/l)	18.93 ± 6.74	16.85(11–33.8)	18.92 ± 6.55	19(2.8–47)	0.786
Prolactin (ngm/dL)	13.68 ± 6.23	11.55(6.3–29.2)	11.04 ± 4.69	9.95(5–29.4)	0.106
Estradiol (ngm/dL)	30.4 ± 10.7	28.5(14–61)	27.6 ± 9.8	26.9(9–62.6)	0.370

**Fig. 3:**
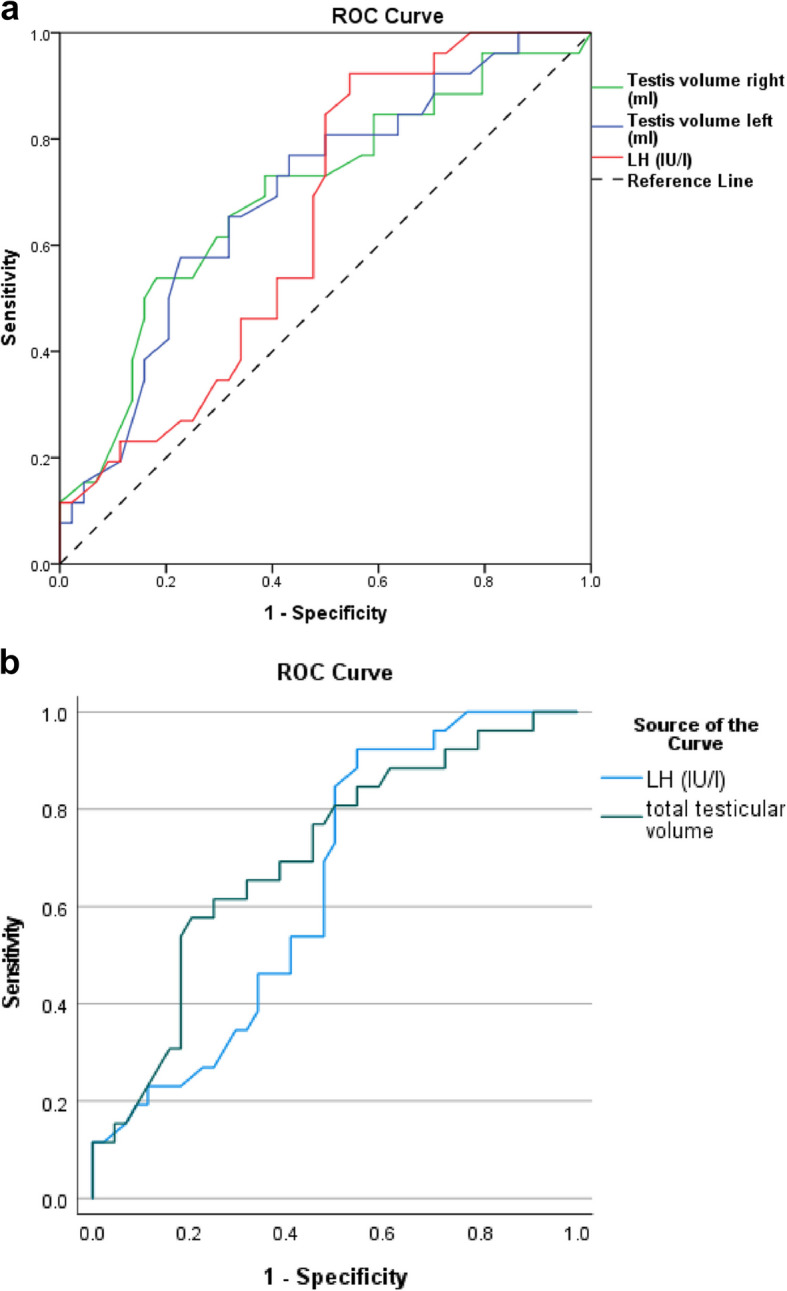
**a**,**b** show ROC curve for predictability of LH and Rt and Lt testicular volumes and total testicular volume for successful sperm retrieval

**Fig. 4:**
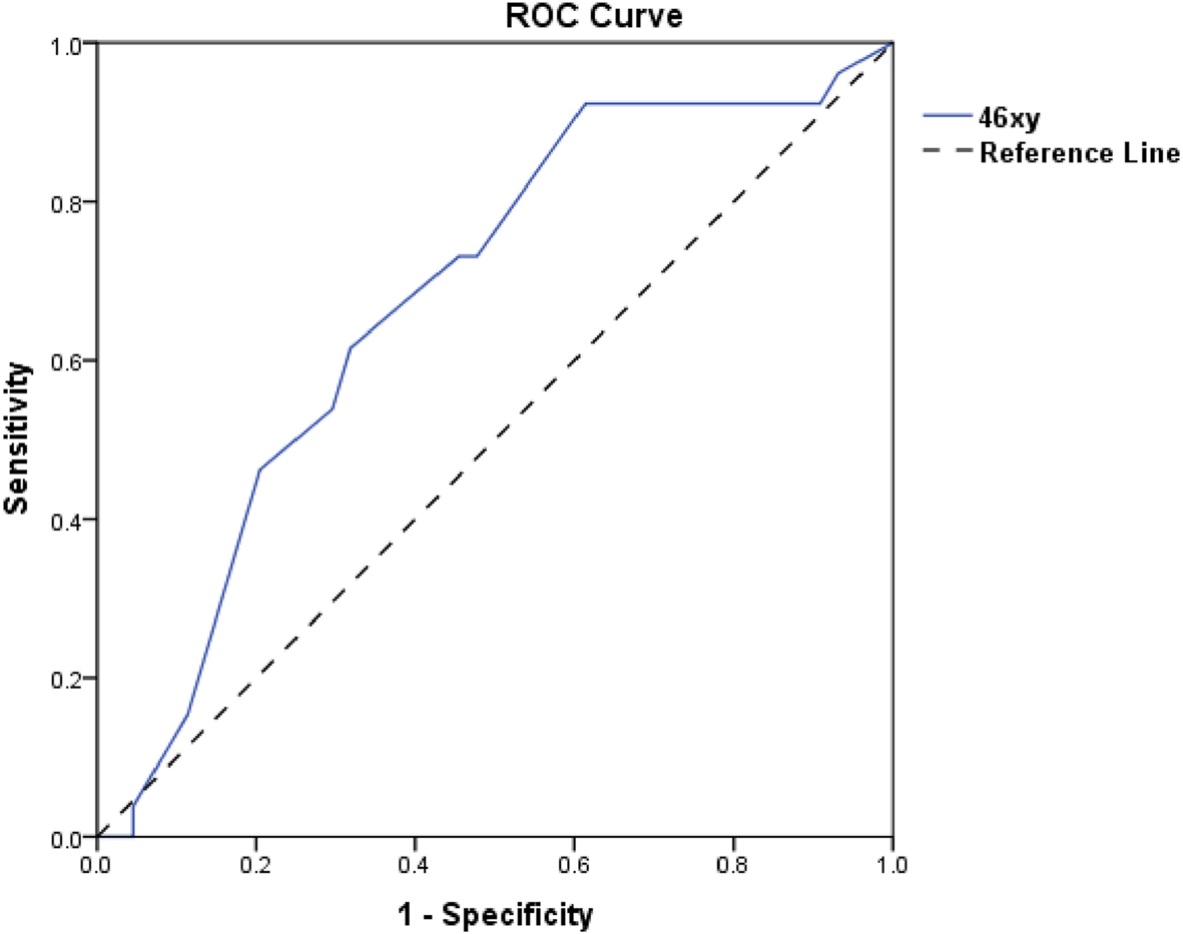
shows ROC curve for predictability of 46xy for successful sperm retrieval

Twenty-six patients out of 50 patients (52%) showed mature sperm in wet preparation, whereas the SRR of the patients with mosaic and non-mosaic KS was (57.1) % and (32.1) %, respectively (Table 2). FISH testing showed that 46xy was significantly higher in the Mosaic genotype (p 0.001). While 47xxy was significantly higher in the pure type (p 0.001) (Table 2). SR was significantly associated with bilateral testicular volumes > 2 ml, total testicular volume > 5 ml and LH < 21.29 IU/L (p 0.007, 0.005 0.044, respectively) (Table 3). FISH testing results showed that higher 46xy and lower 47xxy were significantly associated with successful sperm retrieval (p 0.014, 0.015, respectively) (Table 3). Further analysis demonstrated that receiver operation characteristic (ROC) curve showed Rt and Lt testicular volumes and total testicular volume and LH level and 46xy could significantly predict successful SR with p 0.007, 0.007, 0.005 and 0.044 and 0.015, respectively (Table 4, Figs. 3a,b-4). Moreover, the cutoff point and sensitivity and specificity and positive and negative predictive values for Rt and Lt testicular volumes were as follows 2 ml, 73.1%, 61.4%, 52.78, 79.41, 2 ml, 76.9%, 57.8%, 51.28 and 80.65, respectively (Table 4, Figs. 3 a,b-4). While these values for total testicular volume were as follows 5.255 ml, 61.5, 75, 59.26 and 76.74, respectively (Table 4, Figs. 3 a,b-4). Furthermore, these values for LH and 46xy were as follows 17 IU/l, 73.1%, 50%, 46.34, 75.86, 16.35, 84.6, 50, 50 and 84.6, respectively (Table 4, Figs. 3 a,b-4).

**Table 2:** shows sperms retrieval success rate among participants according to their karyotyping

		Genetic finding by FISH				*P* value
		Mosaic (46XY/47XXY)		Pure (47XXY)		
		Count	%	Count	%	
Sperm retrieval	Negative	6	42.90%	38	67.90%	0.083
	Positive	8	57.10%	18	32.10%	
		Mean ± SD	Median (range)	Mean ± SD	Median (range)	P value
FISH test	46xy	35.9% ± 16.9%	40(8–60) %	17.4 ± 14.3%	12 (0–60) %	0.001
	47xxy	64.1% ± 16.9%	60(40–92) %	82.2 ± 14.3%	88 (40–100) %	0.001

**Table 3:** compares the preoperative factors associated with successful sperm retrieval among the participants

		Sperm retrieval				
		Negative		Positive		*P* value
		Mean ± SD	Median (range)	Mean ± SD	Median (range)	
Age in years		33.1 ± 6	33(22–48)	31.2 ± 6.6	30.5(20–50)	0.136
Testis volume right (ml)		1.95 ± 0.77	1.95(0.7–3.2)	2.54 ± 0.8	2.84(0.7–4.2)	0.007
Testis volume left (ml)		1.97 ± 0.85	1.98(0.66–3.7)	2.59 ± 0.82	2.84(0.92–4.4)	0.007
Total testicular volume (ml)		3.92 ± 1.59	3.81(1.41–6.50)	5.13 ± 1.60	5.69(1.62–8.60)	0.005
TT (nmol/l)		2.8 ± 1.7	2.36(0.14–8.22)	3.83 ± 2.51	3.25(0.98–12)	0.086
FSH (IU/l)		33.57 ± 15.47	32.7(9–75)	35.75 ± 13.36	34.5(11–76)	0.515
LH (IU/l)		17.52 ± 5.79	16.5(2.8–28.5)	21.29 ± 7.15	19.5(13–47)	0.044
Prolactin (ngm/dL)		11.4 ± 4.83	9.95(5.7–29.4)	11.84 ± 5.6	11.87(5–29.2)	0.827
Estradiol (ngm/dL)		26.5 ± 7.6	26.8(9–38)	31 ± 12.8	28(17–62.6)	0.310
FISH test	46xy	17.9 ± 16.5%	10 (0–60)%	26.4 ± 15.3%	25(0–54)%	0.014
	47xxy	81.8 ± 16.3%	90 (40–100)%	73.2 ± 15.5%	75(46–100)%	0.015

**Table 4:** shows ROC analysis for predictability of laboratory and clinical characteristics for successful sperm retrieval

Test Result Variable(s)	AUC	*P* value	Diagnostic indices			Positive predictive value	Negative predictive value	95% Confidence Interval	
			Cutoff	Sensitivity	Specificity			Lower bound	Upper bound
Age	0.393	0.136	-	-	-			0.254	0.532
Right testicular volume (ml)	0.695	0.007	2	73.1%	61.4%	52.78	79.41	0.564	0.825
Left testicular volume (ml)	0.695	0.007	2	76.9%	57.8%	51.28	80.65	0.569	0.822
Total testicular volume	0.703	0.002	5.255	61.5	75	59.26	76.74	0.577	0.829
TT (nmol/l)	0.623	0.087	-	-	-			0.482	0.764
FSH (IU/l)	0.547	0.516	-	-	-			0.411	0.682
LH (IU/l)	0.645	0.027	17	73.1	50	46.34	75.86	0.516	0.773
Prolactin (ngm/dL)	0.516	0.827	-	-	-			0.370	0.662
Estradiol (ngm/dL)	0.573	0.310	-	-	-			0.434	0.712
46xy	0.675	0.008	16.35	84.6	50	50	84.62	0.546	0.805

## Discussion

Our case series study had demonstrated that cases with mosaic KS had shown larger testicular volumes and lower FSH together with higher testosterone level compared to cases with non-mosaic KS. Figure 4.

Consistently, Tsukamoto et al. (2024) had shown similar findings regarding testicular volume, FSH and testosterone levels in their mosaic KS group compared to non-mosaic KS group [18]. Additionally, our case series study showed higher SRR among the mosaic KS group (57.1%) compared to the non-mosaic KS group (32.1%). The underlying reason for this finding could be attributed to the fact that the mean age of both groups was close to each other. This rate is like the chance of finding spermatozoa after micro-TESE in the general population of patients with non-obstructive azoospermia [19, 20]. Contrariwise, Tsukamoto et al. (2024) had revealed comparable SRR from micro-TESE among their groups that could be attributed to the fact that their mosaic KS group was around 14 years older than that of the non-mosaic KS group [18] that affirmed the positive predictive role of aging in these cases. Thus, there was a probability that some men with mosaic KS in their study became azoospermic with aging owing to ejaculating sperm in the ejaculate during their youth [18]. In the same context, Damani et al. (2001) reported successful sperm extraction in a 15-year-old boy [21]. Because spermatogenesis begins before other signs of puberty, they suggested that if the patient had reached maximal sperm production, it might not be necessary to wait until he reached full Tanner stage 5 [21]. Conversely, Okada et al. (2005) stated that testicular sperm extraction should be offered to cases with non-mosaic KS before the critical age of 35 years [22]. Our case series study had shown that the chance of finding spermatozoa was greater in younger patients that could be seen in alignment with these studies. Nevertheless, aging did not show a significant relationship with eventful micro-TESE among cases with mosaic and non-mosaic KS. This finding could be explained by the lack of an association between aging and eventful micro-TESE [23].

Similarly, Van Saen et al. (2012 a,b) failed to report spermatozoa in adolescent boys with KS [24, 25]. Another study conducted by Rohayem et al. (2015) reported lower SRR by micro-TESE in adolescent boys aged 13–14 years [26]. Henceforth, sperm retrieval in young adolescents with KS should not be recommended. Furthermore, a recent review of literature stated that the ideal age for sperm retrieval in KS patients could not be determined [27]. Our case series study demonstrated statistically significant relationships of mosaicism, LH, bilateral testicular volumes and total testicular volume and FISH with successful SR. However, the clinical parameters including FSH, testosterone, prolactin, and E2 levels failed to predict eventful testicular SR. In the same context, a published ESHRE abstract from the 2024 meeting demonstrated an association between SR in KS and testicular volume [28]. In contrast, Boeri et al. (2020) failed to demonstrate any association between clinical, hormonal, and procedural parameters and micro-TESE success among non-mosiac KS together with the necessity for proper counselling of these patients for the high probability of low SSR [29]. Additionally, our case series study had revealed that cases with statistically higher mosaic karyotype (46XY) were associated with higher chances of retrieving spermatozoa. The underlying reason could be attributed to the fact that most men with non-mosaic KS had a small testis together with few numbers of seminiferous tubules [18]. Furthermore, a limited focus of spermatogenesis existed in the seminiferous tubules if any with subsequent low capacity of obtaining sperms through micro-TESE [18]. Conversely, Tsukamoto et al. (2024) stated that their KS cases with eventful micro-TESE had a higher proportion of XY cells in the testis compared to those with uneventful micro-TESE [18].

Noteworthy, all patients with KS who had spermatozoa presented with the mosaic karyotype (XY/XXY) in the testis together with occasional XY spermatogonial cells, because XY germ cells can only complete meiosis and XXY cells are meiotically incompetent [8]. Moreover, testicular spermatozoa obtained from patients with KS were able to induce fertilization, embryo development, and delivery of chromosomally normal children [30, 31]. The risk of transmission of gonosome aneuploidy using spermatozoa from patients with non-mosaic KS is probably not great [30, 31]. Recently, 47XXY spermatogonia demonstrated their potential capability of undergoing meiosis, completing the spermatogenic process, and culminating in the formation of cytogenetically normal spermatozoa [30, 31]. It should be mentioned that a retrospective study stated that pregnancy could be achieved among KS patients (especially mosaic type) by obtaining sperm through micro-TESE together with a normal partner fertility of a high fertilization rate and prescribing appropriate medical treatment prior to micro-TESE [32]. In contrast, it should be mentioned that our patients did not receive any medications prior to micro-TESE and a few patients who were hypogonadal in our case series. However, these patients were prescribed testosterone as a replacement therapy post-procedure. In a similar trend, the value of hormonal therapy to azoopsermic patients prior to micro-TESE is still questionable [33]. Furthermore, patients with Turner syndrome having 46, XX/45X mosaic karyotype showed a higher probability of spontaneous menarche than those with the 45, X non-mosaic karyotype, which was also recognized as the main predictive factor for spontaneous pregnancy [34]. In the same context, patients with mosaic Down syndrome revealed higher IQ scores than those with non-mosaic individuals [35].

Additionally, 7% of adults with mosaic Down syndrome fathered a child, compared with 1% of non-mosaic trisomic probands [36]. In view of the above-mentioned facts, it could be postulated that cases of mosaicism with normal chromosomes could ameliorate the symptoms associated with chromosome aberrations [18].

### Limitations of the study

Admittedly, the small sample size should be mentioned as the major limitation of the study. Another limitation of the study was the inequality of the 2 groups. Nevertheless, it should be mentioned that 14 patients with mosaic KS were recruited out of 70 patients in total. Thus, it represented 20% of our cases that could be seen like the incidence of mosaic KS in general population which represented around 10% to 20% of chromosomal aberrations of KS [5, 21]. It should be mentioned that the real incidence of mosaic KS is under reported due to several reasons. Firstly, chromosomal mosaicism could be found only in the testes, with the normal karyotype of peripheral leukocytes [5]. Secondly, men with mosaic KS might be less severely impacted compared to non-mosaic KS [18]. Thus, men with mosaic KS had higher chances of sperm via ejaculation without the need for being tested to KS [37]. Furthermore, inability to correlate leydig cell hyperplasia nodules count to SRR could be seen as another limitation of the study. Eventually, inability to evaluate the clinical outcomes for these cases regarding the fertilization rate, embryo transfer and clinical pregnancy could be seen as a further limitation. Nevertheless, Tsukamoto et al. (2024) showed that the mosaic KS group had significantly better rates of cleavage and blastocyst development following intracytoplasmic injection compared to the non-mosaic KS group [18]. Thus, it could be stated that the mosaic KS group had a better quality of sperm compared to the non-mosaic KS group [18].

## Conclusion

In our case series study, patients with mosaic KS had a higher rate of successful SR than did those with non-mosaic KS. Mosaicism, LH, testicular volumes and FISH could predict successful SR in these cases.

## Data Availability

No datasets were generated or analysed during the current study.

## Abbreviations

Micro-TESE: Microsurgical testicular sperm extraction
KS: Klinefelter syndrome
LH: Luteinizing hormone
TT: Total testosterone
FSH: Follicle-stimulating hormone
Rt: Right
Lt: Left
SSR: Surgical sperm retrieval
SRR: Sperm retrieval rate
